# Gly460Trp polymorphism of the *ADD1* gene and essential hypertension in an Indian population: A meta-analysis on hypertension risk

**DOI:** 10.4103/0971-6866.64938

**Published:** 2010

**Authors:** P. Ramu, G. Umamaheswaran, D. G. Shewade, R. P. Swaminathan, J. Balachander, C. Adithan

**Affiliations:** Pharmacogenomics Laboratory, Department of Pharmacology, Jawaharlal Institute of Postgraduate Medical Education and Research (JIPMER), Pondicherry - 605 006, India; 1 Department of Medicine, Jawaharlal Institute of Postgraduate Medical Education and Research (JIPMER), Pondicherry - 605 006, India; 2 Department of Cardiology, Jawaharlal Institute of Postgraduate Medical Education and Research (JIPMER), Pondicherry - 605 006, India

**Keywords:** *ADD1*, Essential hypertension, polymorphism, South Indians.

## Abstract

**BACKGROUND:**

Essential hypertension is a complex genetic trait. Genetic variant of alpha adducin (*ADD1*) gene have been implicated as a risk factor for hypertension. Given its clinical significance, we investigated the association between *ADD1* Gly460Trp gene polymorphism and essential hypertension in an Indian population. Further, a meta-analysis was carried out to estimate the risk of hypertension.

**METHODS:**

In the current study, 432 hypertensive cases and 461 healthy controls were genotyped for the Gly460Trp *ADD1* gene polymorphism. Genotyping was determined by real time PCR using Taqman assay. Multiple logistic regression analysis was used to detect the association between Gly460Trp polymorphism and hypertension.

**RESULTS:**

No significant association was found in the genotype and allele distribution of Gly460Trp polymorphism with hypertension in our study. A total of 15 case-control studies were included in the meta-analysis. There was no evidence of the association of Gly460Trp polymorphism with hypertension in general or in any of the sub group.

**CONCLUSIONS:**

We found that the Gly460Trp polymorphism is not a risk factor for essential hypertension in a south Indian Tamilian population. However, the role of *ADD1* polymorphism may not be excluded by a negative association study. Further, large and rigorous case-control studies that investigate gene–gene–environment interactions may generate more conclusive claims about the molecular genetics of hypertension.

## Introduction

Essential hypertension is a highly prevalent, complex, multifactorial disorder that arises from the interaction of genetic predisposition and environmental risk factors. It is significantly associated with the pathophysiology of cardiovascular disorders such as myocardial infarction, stroke, and coronary artery disease. Epidemiologic studies revealed that 20–40% variation in blood pressure is due to genetic heritability.[1,2]

Numerous genetic markers have been identified in the regulation of blood pressure and essential hypertension.[3] One such marker that has drawn substantial attention is α-adducin (*ADD1*) gene. Adducin, a cytoskeleton component, is a heterodimeric protein present in many tissues with α, β and γ subunits involved in cell to cell contact, cell membrane ion transport and signal transduction.[4–6] *ADD1* is one among the proteins that regulate Na^+^-K^+^ ATPase activity. Abnormalities in adducin by genetic mutation have been shown to influence the surface expression and maximum velocity of Na^+^-K^+^ ATPase and subsequently faster renal tubular Na reabsorption.[6] Fifty percent of variation in blood pressure between the Milan hypertensive and normotensive rat strains are due to point mutations in the α and β subunits of adducin.[7] Clinical and experimental studies have demonstrated the potential involvement of *ADD1* in the pathogenesis of essential hypertension both in human and animals.[8]

The gene encoding human *ADD1* is mapped onto the chromosome location 4p16.3. The common molecular variant of the *ADD1* gene causing the substitution of tryptophan instead of glycine (Gly460Trp) at amino acid position 460 was found to be associated with increased risk of hypertension[9] and other cardiovascular risk factors such as hyperlipidemia[10] and left ventricular hypertrophy.[11] There are studies reporting the association between the *ADD1* gene polymorphism and susceptibility to essential hypertension but the results have been inconsistent and inconclusive. Some studies implicated the positive association of the Gly460Trp polymorphism,[12–15] whereas other studies reported negative association.[16–22]

Several studies have described the role of *ADD1* gene polymorphism in hypertension worldwide and there is paucity of data relevant to the Indian population. In view of the above, we carried out a case-control study to investigate the association of *ADD1* gene Gly460Trp polymorphism and susceptibility to essential hypertension in a south Indian Tamil population. In addition, a meta-analysis was conducted to examine the prevalence of Gly460Trp polymorphism comprising 45 studies.

## Materials and Methods

### Study subjects

The study was carried out in 432 unrelated essential hypertensive cases (212 men and 220 women) aged 30-60 years. They were diagnosed and selected from the outpatient clinics of hypertension and internal medicine (JIPMER hospital, Pondicherry, India). All of them were residents of Tamil Nadu and Pondicherry for at least three generations. Patients receiving antihypertensive medications for more than three months (or) newly diagnosed hypertensive patients with systolic blood pressure more than 140mmHg and/or diastolic blood pressure more than 90mmHg (European Society of Hypertension-European Society of Cardiology Guidelines, 2003) on two or more consecutive visits were considered to be hypertensives.[23] The age of hypertension is defined as the time when BP recordings fulfilled the inclusion criteria of hypertension on two consecutive visits before starting the medication or when antihypertensive medication is initiated. Patients with other significant illness that might affect the outcome of investigation, for example, diabetes mellitus, hyperlipidemia, liver or renal disease, congestive heart failure and recent episode of myocardial infarction were excluded. Pregnant and lactating female patients and those receiving medications for other indications that could affect BP were also excluded.

The control group consisted of 461 healthy volunteers (210 men and 251 women) aged 30-60 years. They had no personal or family history of hypertension in the first degree relatives with systolic blood pressure less than 130 mmHg and diastolic blood pressure less than 85 mmHg. Patients who visited the outpatient clinics with minor illness without hypertension, diabetes mellitus, hyperlipidemia and family history of hypertension in previous records were recruited as controls. None in the control group were receiving antihypertensive therapy, treatment for heart disease, or hormone replacement therapy during the time of investigation. Plasma lipid profile and blood glucose level were measured after overnight fasting for both hypertensives and normotensives to rule out diabetes and hyperlipidemia. A detailed family history relating to their pedigree was taken to identify if any of the close relatives of the volunteers or patients were hypertensives. Details such as identification characteristics, body weight and height, drug history were recorded. All the participants were interviewed using a standardized questionnaire with regard to their lifestyle, smoking, alcohol consumption and drug intake. In all subjects, height was measured to the nearest centimeter and weight to the nearest 0.1kg, which was used for calculation of BMI (kg/m^2^ ). Blood pressure was measured by resting the subjects for 10 min in the right arm by using standard sphygmomanometer and the average of three readings taken 2 min apart was recorded. The study was approved by institutional ethics committee and written informed consent was obtained from all the participants.

### Genotyping

Five milliliter of venous blood was collected using ethylene diamine tetra acetic acid (EDTA) as anticoagulant. Genomic DNA was extracted from the peripheral leucocytes using the standard phenol: chloroform method and stored at 4°C. Genotyping for Gly460Trp was carried out by Real Time Thermocycler (ABI Prism 7300, Foster City) using Taqman SNP genotyping assay method. This technique employs fluorogenic 5’nuclease chemistry (also known as Taqman probe-based chemistry) to enable detection of specific PCR product. C__11764545_20 was used as the SNP genotyping assay ID (Applied Biosystems, Foster City). The PCR reaction was carried out in duplicate using 20 µL final volume that contained 10 µL of Taqman Universal PCR master mix (2X), 0.75 µl of 20X working stock of SNP genotyping assay, and 4.5 µL of genomic DNA diluted in DNase free water and 4.75 µL of deionized water. The thermocycler conditions included 1 cycle at 50°C for 2 min; 1 cycle at 95°C for 10 min to activate the AmpliTaq Gold polymerase followed by 40 cycles of denaturation at 92°C for 15 s and annealing/extension at 60°C for 1 min. The allelic discrimination analysis was performed using 7300 SDS software version 1.3.1.

### Statistical analysis

Statistical analysis was done using Statistical Package for Social Sciences software (SPSS windows version release 13, SPSS Inc., Chicago, Illinois, USA). The demographic details of cases and controls with continuous variables were compared using Student’s unpaired ‘t’ test while dichotomous variables were analyzed using Fisher’s exact and Chi square test. Differences in allele frequencies and genotype distribution between hypertensive and normotensive groups were compared by Chi square/Fishers exact test. The association between genotypes and hypertension risk was analyzed by calculating the crude odds ratio (OR) and 95% confidence interval (95% CI) using Chi square/Fishers exact test. The adjusted odds ratio was calculated using unconditional logistic regression and the low risk genotype was designated as the reference category. The observed genotype frequencies were compared with the expected frequencies to check for the Hardy–Weinberg equilibrium and *P* value <0.05 was used as the level of significance.

## Results

### Demographic characteristics of the study subjects

The baseline characteristics of the study subjects are shown in Table 1. The mean age of the controls was higher when compared to cases (44.3 ± 0.4 vs. 47.5 ± 0.4 years, *P*<0.001). No significant difference was observed in the sex distribution, BMI, total cholesterol, triglycerides, LDL and HDL cholesterol levels among the cases and controls. However, there were significant differences in age, systolic blood pressure (SBP), diastolic blood pressure (DBP), smoking pattern, alcohol consumption and VLDL cholesterol levels among the cases and controls. Potential confounding variables that revealed significant differences between cases and controls (age, smoking and alcohol consumption) were taken for logistic regression analysis.

**Table 1:** Demographic details of study subjects

Parameter	Hypertensive cases (*n* = 432)	Controls (*n* = 461)	*P* value
Sex M/F	212/220	210/251	0.3
Age (years)	44.3 ± 0.4	47.5 ± 0.4	0.001
Body mass index (kg/m^2^)	23.0 ± 0.3	22.9 ± 0.2	0.7
Systolic blood pressure (mmHg)	153.4 ± 0.8	117.5 ± 0.5	0.0001
Diastolic blood pressure (mmHg)	97.0 ± 0.5	78.2 ± 0.3	0.0001
Alcoholics N (%)			
Never	317 (73.4)	368 (79.8)	0.03
Current	115 (26.6)	93 (20.2)	
Smokers N (%)			
Never	359 (83.1)	410 (88.9)	0.01
Current	73 (16.9)	51 (11.1)	
Total cholesterol (mg / dL)	176.0 ± 1.5	172.7 ± 1.7	0.1
Triglycerides (mg / dL)	120.8 ± 2.9	118.3 ± 2.5	0.1
HDL cholesterol (mg / dL)	40.8 ± 0.4	41.0 ± 0.5	0.8
LDL cholesterol (mg /dL)	111.5 ± 1.3	109.2 ± 1.5	0.5
VLDL (mg / dL)	24.8 ± 0.6	23.1 ± 0.5	0.03

Values in parenthesis indicate percentage; values are mean ± SEM and numbers

### Genotype distribution of ADD1 (Gly460Trp) gene polymorphism among cases and controls

The genotype and allele frequencies are shown in Table 2. No significant differences were found in the genotype and allele distribution between the cases and controls. The frequency of Gly460Trp genotypes Gly460Gly, Gly460Trp and Trp460Trp were 59.1, 35.6, 5.3, and 63.6, 32.3, 4.1% in cases and controls, respectively. The frequency of the variant allele 460Trp among the cases and controls were 23 and 20%, respectively (OR 1.2; 95% CI 0.9-1.5, *P*>0.05).

**Table 2:** Genotype and allele distribution of *ADD1* (Gly460Trp) gene polymorphism among hypertensive cases and controls.

Gly460Trp	Cases (*n* =432)	Controls (*n*=461)	OR (95% CI)	*P* value	OR^a^ (95% CI)	*P* value
Genotype						
Gly/Gly	255 (59.1)	293 (63.6)	1.0	Ref	1.0	Ref
Gly/Trp	154 (35.6)	149 (32.3)	1.2 (0.9 -1.6)	0.2	1.1 (0.8 -1.6)	0.3
Trp/Trp	23 (5.3)	19 (4.1)	1.4 (0.7 - 2.6)	0.3	1.3 (0.8 - 3.0)	0.3
Allele						
Gly	0.77	0.80	1.2 (0.9-1.5)	0.2		
Trp	0.23	0.20				

Values in parenthesis indicate percentage

a Odds ratio according to genotype was estimated after adjusting the cofounding variables for age, smoking and alcohol consumption

### Gender specific distribution of ADD1 (Gly460Trp) gene polymorphism among cases and controls

Gender specific analysis was carried out by comparing hypertensive men and women with their respective control groups as shown in Table 3. The baseline characteristics of male and female cases were compared with their respective control groups (Table not shown). Age was higher in female cases when compared to female controls (42.6 ± 0.5 *vs*. 46.8 ± 0.5, *P*<0.02). Smokers (34% *vs*. 24%, *P*<0.001) and alcoholics (54% *vs*. 44%, *P*<0.001) were higher in male cases as compared to male controls. These confounding variables were adjusted by logistic regression analysis to calculate the adjusted odds ratio as shown in Table 3. Gender specific distribution also did not reveal any significant association before and even after adjusting the confounding variables among the cases and controls.

**Table 3:** Gender specific distribution of *ADD1* (Gly460Trp) gene polymorphism among cases and controls

Gly460Trp	Male						Female					
	Cases (*n* = 212)	Controls (*n* = 210)	OR (95% CI)	*P* value	OR^a^ (95% CI)	*P* value	Cases (*n* = 220)	Controls (*n* = 251)	OR (95% CI)	*P*	OR^a^ (95% CI)	*P* value
Genotype												
Gly/Gly	126 (59.4)	139 (66.2)	1.0	Ref	1.0	Ref	129 (58.6)	154 (61.4)	1.0	Ref	1.0	Ref
Gly/Trp	75 (35.4)	65 (31.0)	1.3(0.8-1.9)	0.3	1.2 (0.8-1.8)	0.4	79	84 (33.5)	1.1 (0.8-1.7)	0.6	1.1 (0.8-1.8)	0.5
Trp/Trp	11 (5.2)	6 (2.8)	2.0(0.7-5.6)	0.2	2.0 (0.7-5.7)	0.2	(35.9)	13 (5.2)	1.1 (0.4-2.4)	0.9		0.9
Allele							12 (5.5)				1.1 (0.5-2.6)	
Gly	0.77	0.82	1.3(0.9-1.8)	0.1			0.77	0.79	1.1(0.8-1.5)	0.6		
Trp	0.23	0.18					0.23	0.21				

Values in parenthesis indicate percentage

a Odds ratio according to genotype was estimated after adjusting the cofounding variables for smoking and alcohol consumption in male subjects and for age in female subjects.

### Prevalence of 460Trp allele among different ethnics

A meta-analysis was conducted on Gly460Trp polymorphism of the *ADD1* gene comprising 45 studies[9–11,13–22,24–55] among different populations. In addition to the overall analysis, subgroup analysis for each ethnicity was also performed to find out the allelic prevalence. Ethnicity was categorized into three main groups: (1) Caucasians, (2) Asians and (3) Blacks. The overall prevalence of the 460Trp allele in 37640 subjects amounted to 31.9%. The Gly460Gly, Gly460Trp and Trp460Trp genotype frequencies were 49.7%, 36.7% and 13.6% respectively. The prevalence of the 460Trp allele was higher in Asians (55.7%), followed by Caucasians (20.9%), and lower in blacks (6.5%).

### 460Trp allele and hypertension

Further, we also analyzed the association between *ADD1* Gly460Trp polymorphism and essential hypertension by combining 15 case-control studies, involving 3187 cases and 3720 controls[9,13–22,24–27] as shown in Table 4. Surprisingly, the overall prevalence of 460Trp allele was higher in control subjects that amounted to 34.7% as compared to 32% in cases. The estimated overall risk for hypertension associated with 460Trp allele was not significant with odds ratio lesser than 1.0 (OR 0.9; 95% CI 0.8-0.9). Sub-group analysis by ethnicity showed lack of association in Caucasians (OR 0.9; 95% CI 0.8-1.0), Asians (OR 1.1; 95% CI 0.9-1.2) and Blacks (OR 1.1; 95% CI 0.8-1.5) as well. However, the odds of risk was significantly higher in a study conducted in European Caucasians (OR 1.6; 95% CI 1.2-2.0), Japanese (OR 1.5; 95% CI 1.1-2.1) and Africans (OR 2.7; 95% CI 1.1-6.7).[9,13,14] In the present meta-analysis the Gly460Trp polymorphism does not predict the risk of essential hypertension.

**Table 4:** Risk of hypertension in Caucasian, Asian and Black subjects as a function of Gly460Trp genotype.

First author	Case/Control					OR (95% CI)		
	Genotype			Allele				
	Gly460Gly	Gly460Trp	Trp460Trp	Gly460	Trp460	GG vs.GT	GG vs.TT	G vs.T
Caucasians								
Clark, 2000[25]	88/74	36/44	4/10	212/192	44/64	0.7 (0.4-1.2)	0.3 (0.1-1.1)	0.6 (0.4-0.9)
Melander, 2000[26]	257/259	107/138	10/22	621/656	127/182	0.8 (0.6-1.0)	0.4 (0.2-1.0)	0.7 (0.6-0.9)
Cusi, 1997[9]*	289/243	166/78	22/11	744/564	210/100	1.8 (1.3-2.5)	1.7 (0.8-3.5)	1.6 (1.2-2.0)
Kamitani, 1998[17]	43/38	25/31	6/8	111/107	37/47	0.7 (0.3-1.4)	0.6 (0.2-2.0)	0.7 (0.4-1.2)
Wang, 1999[24]	70/112	33/73	9/11	173/297	51/95	0.7 (0.4-1.2)	1.3 (0.5-3.3)	0.9 (0.6-1.3)
Alam, 2000[27]	51/84	31/35	3/5	133/203	37/45	1.4 (0.8-2.6)	1.0 (0.2-4.3)	1.2 (0.8-2.0)
Mead, 2005[22]	51/145	20/82	2/15	122/372	24/112	0.4 (0.1-1.7)	0.7 (0.4-1.2)	0.6 (0.4-1.0)
Total Asians	849/955	418/481	56/82	2116/2391	530/645	1.0 (0.8-1.1)	0.8 (0.5-1.0)	0.9 (0.8-1.0)
Ishikawa, 1998[16]	33/35	85/96	52/63	151/166	189/222	0.9 (0.5-1.6)	0.9 (0.5-1.6)	0.9 (0.7-1.2)
Kato, 1998[18]	44/30	109/66	70/63	197/126	249/192	1.1 (0.6-1.9)	0.7 (0.4-1.3)	0.8 (0.6-1.1)
Tamaki, 1998[13]*	13/26	76/70	47/32	102/122	170/134	2.1 (1.0-4.5)	2.9 (1.3-6.5)	1.5 (1.1-2.1)
He, 2000[20]	35/39	73/53	30/29	143/131	133/111	1.5 (0.8-2.7)	1.1 (0.6-2.3)	1.0 (0.8-1.5)
Ju, 2003[15]	57/109	109/248	90/135	223/466	289/518	0.8 (0.6-1.2)	1.2 (0.8-1.9)	1.1 (0.9-1.4)
Shin, 2004[21]	52/95	147/283	122/204	251/473	391/691	0.9 (0.6-1.4)	1.0 (0.7-1.6)	1.0 (0.9-1.3)
Total Blacks	234/334	599/816	411/526	1067/1484	1421/1868	1.0 (0.9-1.3)	1.1 (0.9-1.4)	1.1 (0.9-1.2)
Barlassina, 2000[14]*	126/88	20/6	2/0	272/182	24/6	2.3 (0.9-6.0)	3.5 (0.2-73.8)	2.7 (1.1-6.7)
Larson, 2000[19]	408/374	63/54	1/4	879/802	65/62	1.0 (0.7-1.6)	0.2 (0.02-2.0)	0.9 (0.6-1.4)
Total	534/462	83/60	3/4	1151/984	89/68	1.2 (0.8-1.7)	0.6 (0.1-3.0)	(0.8-1.5)
All subjects	1617/1751	1100/1357	470/612	4334/4859	2040/2581	0.9 (0.8-1.0)	0.8 (0.7-0.9)	0.9 (0.8-0.9)

* Studies showed significant risk for hypertension

## Discussion

Cardiovascular disease is the leading cause of death in both the economically developed and developing countries. The recent epidemiological investigation on Indian population has shown that the incidence of cardiac events and cardiac mortality would be doubled by the year 2020. In India, hypertension directly accounts for about 57% of stroke and 27% of CAD deaths.[56]

To the best of our knowledge, the present study is the first and largest case-control study to address the role of Gly460Trp polymorphism of the *ADD1* gene and susceptibility to essential hypertension in an Indian population. Our data showed that the genotype and allele frequencies were similar between hypertensive cases and controls and were not associated with hypertension.

Despite the difference in the variant allele frequency, the results of the present study were in accordance with other studies conducted in Japanese, Chinese, Koreans and Caucasians.[16,18,20,21,24] In contrast, the results were not in agreement with those published from the same Caucasians, Japanese, Africans and Chinese populations.[9,13–15] When the influence of Gly460Trp polymorphism was analyzed for male and female participants separately, differences in genotypes and alleles were statistically insignificant and were not associated with hypertension. Very few studies investigated the gender specific association between Gly460Trp polymorphism and hypertension. A case-control study in a Japanese population showed significant association between Gly460Trp polymorphism and hypertension in female hypertensive cases.[49]

Significant association between *ADD1* Gly460Trp gene polymorphism and essential hypertension was first reported by Cusi *et al*, in white population. They observed that the Gly460Trp polymorphism is associated with salt sensitive form of hypertension.[9] Later on, several other studies were carried out to establish the role of *ADD1* Gly460Trp polymorphism in the etiology of essential hypertension. However, varied results have been obtained.[12–22]

It has been postulated that *ADD1* may affect blood pressure by modulating renal tubular reabsorption of sodium through the activation of Na^+^, K^+^, -ATPase with the *ADD1* 460Trp exhibiting higher affinity for the Na^+^, K+, -ATPase pump. Several studies reported the relationship between *ADD1* polymorphism and anti-hypertensive drug response.[9,44,57,58] Specifically, carriers of 460Trp variant showed a greater fall in mean arterial BP and lower plasma renin activity than Gly460Gly homozygous wild type carriers after hydrochlorothiazide treatment in Caucasians.[9,44,57] On the other hand, similar results were not observed in another Caucasian population after treatment with diuretics, ACE inhibitors and β blockers.[58] However, in a Caucasian population gene–gene–drug interaction (variants of *ADD1* W allele + *AGT* T allele + diuretics) significantly reduced DBP in hypertensive cases after diuretic therapy.[58]

In the Rotterdam study, the *ADD1* polymorphism predicted the high risk of mortality in type 2 diabetic patients and increased mean common carotid IMT in relation to hypertension.[28] Furthermore, this polymorphism was also found to be associated with atherosclerosis, stroke and MI.[58] Similarly, 460Trp allele was associated with stroke in Dutch women and the risk was elevated in the presence of systolic hypertension.[29] In contrast, it was not associated with MI in Dutch men.[30] A large prospective study conducted in middle aged men suggested that the variant allele 460Trp was significantly associated with peripheral artery disease and coronary artery disease.[31] In addition, carriers for 460Trp allele were more sensitive to changes in body sodium than the carriers for Gly460 in hypertensives, suggesting an increased reabsorption of sodium in the proximal tubule.[32] Factors that contribute to this difference may include genetic diversity within the subgroup, different study designs, sample size and environmental exposures.

Even though several studies were done on *ADD1* polymorphism, meta-analysis is not available so far. In the present meta-analysis, the occurrence of variant allele 460Trp was higher in Asians and lower in Africans, a trait that did not match with other ethnic groups. In addition, the consistency of genetic effects across populations from different ethnicities was also investigated. Race was a major determinant of the Gly460 and 460Trp allele frequencies. The overall lack of association between Gly460Trp *ADD1* gene polymorphism and hypertension and the discrepancy of results between Caucasians, Asians and blacks might be because of other unidentified polymorphisms that exist in the *ADD1* gene that affect the susceptibility to hypertension or other loci that might be in linkage disequilibrium with the examined polymorphism of *ADD1* gene. Rather than the individual mutation, interaction of multiple polymorphisms or haplotypes can be a major determinant of disease susceptibility. Finally, the limitation of the meta-analysis is that our results were extracted directly from published articles and not from the original data provided by the authors.

The strength of our study includes strict selection of cases and controls from homogenous population. In conclusion, neither the present study nor the meta-analysis offers evidence for the *ADD1* variant 460Trp in the causation of essential hypertension suggesting that it is unlikely to be a predisposing factor in hypertension.
